# Profiles of sleep disturbances in Angelman, Prader-Willi, and Rett syndromes: analysis of standardized questionnaires

**DOI:** 10.3389/fneur.2026.1854819

**Published:** 2026-07-22

**Authors:** Lindsay M. Oberman, Olivia J. Veatch, Sarika U. Peters, Walter E. Kaufmann

**Affiliations:** 1 National Institute of Mental Health Intramural Research Program, National Institutes of Health, Bethesda, MD, United States; 2 Division of Medical Informatics, Department of Psychiatry and Behavioral Sciences, University of Kansas Medical Center, Kansas, KS, United States; 3 Vanderbilt Kennedy Center, Department of Pediatrics, Vanderbilt Kennedy Center, Vanderbilt University Medical Center, Vanderbilt University, Nashville, TN, United States; 4 Department of Human Genetics, School of Medicine, Emory University, Atlanta, GA, United States; 5 Department of Neurology, Boston Children’s Hospital, Boston, MA, United States

**Keywords:** Angelman syndrome, pediatric sleep, Prader-Willi syndrome, Rett syndrome, sleep questionnaires

## Abstract

**Introduction:**

Individuals with neurodevelopmental disorders (NDDs) are at increased risk of having sleep difficulties. A variety of sleep problems have been reported in Angelman syndrome (AS), Prader-Willi syndrome (PWS), and Rett syndrome (RTT). The present study intended to expand an earlier characterization of sleep difficulties in a large AS, PWS, and RTT sample by analyzing subscales of standardized sleep questionnaires.

**Methods:**

Scores from children (2–18 years) with AS (*n* = 74), PWS (*n* = 90) or RTT (*n* = 241) on components of the Children’s Sleep Habits Questionnaire (CSHQ), the Sleep-Related Breathing Disorder (SRBD) scale, and the Pediatric Daytime Sleepiness Scale (PDSS) were compared between NDDs and with those from a group of neurotypical siblings (*n* = 282). Additional comparisons of scores after a 12-month follow-up evaluation, in a subset of individuals, were also performed. Data were analyzed using nonparametric tests and, for changes over time, both cross-sectionally and longitudinally.

**Results:**

Comparisons with neurotypical children showed that night waking and snoring were increased in the three NDDs while parasomnias and daytime sleepiness only in AS and RTT. Children with RTT also had the highest scores on measures of disordered breathing. At the 12-month follow-up, scores decreased in neurotypical children but had variable courses in the NDDs, with increased disordered breathing scores characterizing AS and RTT. There was high agreement among disordered breathing measures, but not among daytime sleepiness scales. Overall, CSHQ scores were relatively stable within NDDs.

**Conclusion:**

Sleep questionnaires revealed disorder-specific profiles of sleep problems that could assist in their identification and management. The CSHQ and the SRBD, including their subscales, appear to be consistent measures particularly for sleep-disordered breathing and, therefore, suitable for clinical and research use in severe NDDs. Follow-up studies should expand the range of instruments to include objective measures in the characterization of sleep abnormalities in AS, PWS, and RTT.

## 1 Introduction

Sleep difficulties are among the most common clinical manifestations in neurodevelopmental disorders (NDDs) (1). Prevalence of sleep problems is higher in individuals with NDDs than in neurotypicals, with estimates of 50–95% in NDD populations compared to 25% in neurotypical populations (2–5). Although the range of prevalence estimates in NDDs may be explained by methodological factors, frequency and other features of different sleep problems seem to be disorder-specific (1, 6). For example, in their meta-analysis of 19 genetic NDDs, Agar and colleagues reported that prevalence of sleep-related breathing difficulties ranged from 2% in Angelman syndrome (AS) and Jacobsen syndrome to over 70% in mucopolysaccharidoses while excessive daytime sleepiness ranged from 9% in Jacobsen syndrome to 60% in Smith–Magenis syndrome (7). Considering that sleep problems are associated with poorer cognitive and behavioral outcomes in the general population and NDDs (1, 5, 8–19), characterizing the most common types of sleep difficulties in a particular disorder may lead to their earlier identification and to more targeted treatments (1, 4–7). Underscoring the importance of delineating sleep difficulties in NDDs, a recently organized a workshop on Neurodevelopmental Disorders and Sleep was by the National Heart, Lung, and Blood Institute (NHLBI) at the National Institutes of Health (NIH) to identify research gaps and opportunities in the field. The conclusions of the event included limited availability of mechanistic data and use of objective diagnostic methods. They also highlighted the correlation between sleep disturbances and more severe NDD symptoms (National Heart, Lung, and Blood Institute (NHLBI)/National Institutes of Health (NIH) Neurodevelopmental Disorders and Sleep Workshop (May 15–16, 2025): www.nhlbi.nih.gov/events/2025/neurodevelopmental-disorders-and-sleep-workshop). A NIH-funded Rare Diseases Clinical Research Network (RDCRN) project on three severe NDDs linked by epigenetic mechanisms, Angelman syndrome (AS), Prader-Willi syndrome (PWS), and Rett syndrome (RTT), has recently characterized sleep disturbances in a large sample of affected individuals (20), the findings have implications for severe NDDs in general.

AS is a rare genetic disorder affecting both sexes and with a prevalence of 1 in 12,000–24,000 (21, 22). It is associated with a loss in the ubiquitin protein ligand E3A (UBE3A), key for protein homeostasis, due most commonly to a deletion of the maternal chromosome 15q11-q13 region containing the maternally imprinted *UBE3A* gene (only the maternal copy is active in neurons) (22–24). AS is characterized by severe global developmental delay/severe intellectual disability, absent or minimal spoken language, seizures, happy demeanor and outbursts of laughter, gross and fine motor deficits, tremors, hyperactivity, stereotypical behavior, anxiety, gastrointestinal problems, and sleep disturbances (20, 22–26). PWS affects both males and females, with overall prevalence estimates of 1 in 10,000–30,000 births (22). It is associated, in most cases, with a loss due to a deletion of the paternally imprinted chromosome 15q11-q13 region. In contrast to AS, PWS is not linked to the loss of function of *UBE3A* but of other genes in the region (e.g., *SNORD116*) (22, 24, 27). PWS’ phenotype is characterized earlier by developmental delay/mild–moderate intellectual disability, hypotonia, and feeding difficulties, which is followed in childhood by hyperphagia that is frequently associated with early-onset morbid obesity, and sleep-disordered breathing (20, 22, 24, 27, 28). RTT occurs predominantly in females, with a prevalence of 1 in 10,000–23,000 (29), with most cases linked to loss-of-function variants in the X-linked *MECP2* gene that encodes the transcriptional regulator methyl-CpG-binding protein 2 (*MECP2*) (29). RTT is characterized by developmental regression following an initial period of apparently typical development. Loss of spoken language and purposeful hand use, stereotypical hand movements, and impaired ambulation constitute RTT’s core diagnostic features (30, 31). The regression phase is followed by a period of relative stabilization when severe cognitive impairment and other clinical manifestations become evident, including seizures, growth failure, gastroesophageal symptoms (e.g., constipation), scoliosis, breathing and autonomic dysfunction, behavioral abnormalities, and sleep problems (29).

Although broadly linked by epigenetic mechanisms, each of the aforementioned NDDs involves different genes and signaling pathways. Therefore, it is not surprising that they present with different clinical manifestations including disorder-specific sleep abnormalities. In AS, sleep disturbances such as difficulties initiating and maintaining sleep, sleep terrors, sleep-related seizures, disorientation when aroused, snoring, teeth grinding, bed wetting, sleepwalking, and circadian rhythm abnormalities have been reported (1, 6, 25, 32–34). Studies from AS mouse models have shown mixed results; one found that circadian rhythms were intact, but abnormal sleep patterns arose from a deficit in accumulation of sleep drive (i.e., disruption to sleep homeostasis) (35). In contrast, in a different study Shi et al. (2015) reported alterations to circadian rhythms characterized by a longer circadian period that leads to delayed phase, which they hypothesize could account for sleep onset latency and shorter sleep duration in individuals with AS (36). Melatonin deficits have also been implicated in the pathogenesis of sleep disturbances in AS (37). In PWS, distinctive sleep problems include obstructive and/or central sleep apnea, hypersomnia (not always in the presence of sleep-disordered breathing), and occasionally narcolepsy (1, 6, 38–43). Numerous physiological and pathological factors may contribute to different types of sleep disruption in PWS. For example, sleep disordered breathing may relate to physiological characteristics (e.g., obesity, hypotonia, craniofacial restrictions) and/or hypothalamic dysfunction which are all core features of PWS (44). Hypothalamic dysfunction could also underly expression of narcolepsy in PWS due to reduced orexin production from hypothalamic neurons (44). In addition, as in AS, circadian abnormalities mediated by the interaction between genes encoded in the PWS critical region with core circadian clock genes have been observed. These include *MAGEL2*, which inhibits two sleep-promoting genes (i.e., *CLOCK* and *BMAL1*), and *SNORD116*, which may influence expression of the *CLOCK* gene (45–47). As such, lack of expression of *MAGEL2* and *SNORD116* in PWS may result in issues with daytime sleepiness and mistimed sleep. In RTT, a wide range of sleep and circadian rhythm problems, including difficulties initiating and maintaining sleep, reduced REM sleep, and sleep-disordered breathing, have been reported (1, 15, 19, 48–52). Because of its high frequency, impaired sleep pattern is considered a supportive criterion for the diagnosis of RTT (30). Studies of RTT mouse models suggest that the underlying etiology of sleep/wake problems in RTT is the role that MeCP2 plays in the circadian timing system, with disruptions to the circadian clock within the suprachiasmatic nucleus (SCN), where MeCP2 is highly expressed (53, 54).

The RDCRN project confirmed and expanded the literature by showing that a high proportion of individuals across all three AS, PWS, and RTT presented with clinically significant sleep problems (20). Overall sleep problems, as measured by total scores on the Children’s Sleep Habits Questionnaire (CSHQ) (55, 56), were present at a clinical level in 70–95% of children with these NDDs (i.e., scores ≥ 41). Moreover, using the Pediatric Sleep Questionnaire/Sleep-Related Breathing Disorder scale (PSQ/SRBD) (57) total scores, we found that approximately half of children with AS, PWS, and RTT, but not their neurotypical siblings, had clinically significant sleep-related breathing difficulties. Other findings included higher CSHQ total scores in AS than in PWS or RTT, greater daytime sleepiness (i.e., Pediatric Daytime Sleepiness Scale [PDSS] scores) (58) in older children with RTT than in AS or PWS and higher total scores on the SRBD in RTT than in PWS, the latter previously associated with sleep-related breathing problems (38–40). Notably, the initial publication on this sleep study did not include analyses of the longitudinal data that were collected at a 12-month follow-up for a subset of participants (20).

Given the literature and the initial findings from the RDCRN sleep investigation and the need for further delineating NDD-specific sleep abnormalities in order to improve their diagnosis and management, a more fine-grained investigation was warranted. Specifically, our early analyses did not include subscales or individual components of the CSHQ and the SRBD, which could have revealed additional NDD-specific profiles or longitudinal data that may have delineated the evolution of sleep difficulties (20). These additional data are essential for implementing early diagnosis and adequate follow ups as well as for selecting outcome measures for clinical trials. Consequently, The present investigation extended the prior analyses by comparing CSHQ and SRBD subscales and PDSS scores from affected individuals with those from unaffected siblings and among the three NDDs, and evaluated changes in scores over a 12-month period.

## 2 Materials and methods

### 2.1 Participants

Participants were recruited from the RDCRN natural history studies for AS (NCT00296764), PWS (NCT03718416), and RTT (NCT02738281), a NIH initiative linking three NDDs associated with epigenetic abnormalities, at the following consortium sites: University of Alabama at Birmingham (RTT), Baylor College of Medicine (AS, RTT), Boston Children’s Hospital (AS, RTT), Greenwood Genetic Center (AS, RTT), Rady Children’s Hospital San Diego (AS), University of California, Irvine (PWS), University of Florida (PWS), University of Kansas Medical Center (PWS), and Vanderbilt University (AS, PWS). Criteria for inclusion in the AS, PWS, and RTT natural history studies have been reported in previous publications (26, 28, 31) and included in the Supplementary Material. Data were also obtained from unaffected siblings of the participants as a comparison group. These individuals were neurotypical and did not have any genetic, neurological, or behavioral disorder. Inclusion of the comparison group was based on the need to control for other variables potentially influencing the study’s outcome, specifically general genetic and environmental background shared by affected children and their siblings. Moreover, since the sleep questionnaires were mainly validated in neurotypical individuals such group provides a reference for interpreting scores in children with NDDs.

Requirements for enrollment in this sleep study included to be 18 years old or younger, and to live in a private home with a caregiver familiar with the child’s individual sleep habits, who was fluent in English (i.e., at the time of the study, sleep questionnaires were only available in English). Data on 20 individuals older than 18 years were also collected by error. This provided the opportunity for conducting secondary analysis of a group of 15 adults with RTT. Data from two adult participants, each with AS or PWS, and from 1 unaffected adult sibling, were not analyzed because of the small numbers. Affected participants, ≤ 18 years, were defined as individuals with a diagnosis of AS (*n* = 74), PWS (*n* = 90), RTT (*n* = 241), or neurotypical siblings of any of the affected participants. The study included two assessments within a 12-month period. The following subset of participants had a second evaluation: Neurotypical (*n* = 132), AS (*n* = 30), and RTT (*n* = 139). Only one participant with PWS had a follow-up assessment and as such were not included in analyses investigating score changes over time.

### 2.2 Procedures

The RDCRN natural history studies collected a wide range of data (i.e., diagnosis, genetics, medical history, clinical features and evolution) on affected individuals as well as unaffected siblings. In this sleep study, participants enrolled in the main protocols were administered a set of sleep questionnaires after obtaining specific informed consent, following institutional review board approval. For each participant, a caregiver who was familiar with the child’s sleep pattern completed each of the study questionnaires. The latter were checked for completeness and caregivers were requested to complete unfinished items, if needed. Since the study was designed to cover a wide range of sleep problems, three questionnaires were used to ensure their comprehensive assessment. The age inclusion criteria were based on the ranges validated for each instrument, which allowed comparisons with neurotypical subjects as well as clinical interpretation of scores using reference cut-offs (55–57).

We analyzed data from the three following questionnaires:

1. Children’s Sleep Habits Questionnaire (CSHQ) is a retrospective, 33-item parent/caregiver questionnaire that has been validated and widely used to evaluate sleep behavior in young children, from age 2 to 10 years (55, 56). It includes additional items dealing with timing and amount of sleep and night awakenings. Questions are answered in terms of frequency (rarely, sometimes, usually) and whether the item is a problem or not (yes, no). A total score is calculated by adding the 33 questions, with higher scores indicating more problematic sleep behaviors. Scores can range from 33 to 99 with scores of 41 or more suggested to represent a clinically significant problem (32). Items are grouped into 8 subscales: Bedtime resistance, Sleep onset delay, Sleep duration, Sleep anxiety, Night waking, Parasomnias, Sleep disordered breathing, and Daytime sleepiness.
2. Sleep-Related Breathing Disorder (SRBD) scale, also termed Pediatric Sleep Questionnaire (PSQ) (57), is a screening tool for sleep disordered breathing, widely used in research and clinical settings, which can assist clinical decision making when polysomnography is unavailable (59). The SRBD includes 22 closed response question items, comprising three symptom complexes (subscales): Snoring, (excessive) Daytime sleepiness, and Inattentive or hyperactive behavior. Total scores for the SRBD are based on the mean response to all relevant questions where “yes” = 1 and “no” = 0, with a score of 0.33 or constituting greater evidence of sleep disordered breathing. For the subscales, scores were calculated by simply adding the response scores. The SRBD was validated for ages 2 to 18 years against polysomnography (59). We analyzed SRBD total and subscale scores.
3. Pediatric Daytime Sleepiness Scale (PDSS) includes 8 questions that assess daytime sleepiness and are similar to the Epworth Sleepiness Scale for adults (35). The range of possible scores is 0 to 32, and no cut-off score has been established for this questionnaire. The PDSS has been validated for ages 5 to 17 years (9, 13, 58). Since the PDSS does not have different components, we only analyzed total scores.

### 2.3 Analyses

Primary analyses compared total and subscale scores, where appropriate, between the four groups: Neurotypicals, AS, PWS, and RTT. Two sets of secondary analyses were performed. The first was a comparison of CSHQ, SRBD, and PDSS scores within each of the four groups for those individuals with two time points (i.e., serial analysis). A second secondary analysis compared cross-sectionally scores on the three questionnaires between two age groups within each NDD and in Neurotypicals: younger children (2–10 years) versus older children and adolescents (10–18 years). These age ranges were based on the validated age range for the CSHQ (2–10 years), the most widely applied sleep questionnaire in pediatric populations (55, 56). In the case of RTT, additional within-disorder comparisons with a group of adults (18 years and older) were also possible thanks to the unplanned recruitment of these individuals. These cross-sectional analyses intended to explore the stability of scores during two major developmental periods and complement, in this way, the longitudinal evaluation that examined only two time points within a relatively short interval. Another goal was to evaluate the potential adequacy of the CSHQ in individuals older than 10 years.

Age was determined by calculating the difference between the birthdate and date of the visit when the caregiver completed the sleep questionnaires. For a subset of individuals (*n* = 57), the birthdate was missing or the calculated age at the visit was improbable (i.e., negative or less than 1 year). In these cases, ages were replaced by the reported age at registration when available. When age could not be determined, individuals were excluded. In addition, to comply with the validity of the questionnaires, individuals who were younger than 2 years were excluded, and those older than 18 years were included only if they had the diagnosis of RTT.

Age and questionnaire score distributions were assessed by histograms and Shapiro–Wilk tests. Due to the lack of normality, nonparametric tests were conducted for all analyses. Comparisons of baseline scores between the four groups (primary analysis) employed Kruskal-Wallis tests followed by Bonferroni-adjusted *post-hoc* tests. Within group longitudinal (serial) analyses employed Wilcoxon Signed Rank tests while cross-sectional comparisons between younger children and older children and adolescents, within each NDD and in the Neurotypical group, were performed with the Wilcoxon Sum Rank (Mann–Whitney U) test. Primary analyses were also conducted with Benjamini-Hochberg adjusted Mann–Whitney U tests to complement multiple comparison Bonferroni corrections. Correlation analyses between items of Daytime sleepiness scales were done using the Spearman correlation test.

Descriptive data are presented as mean, standard deviation, and range for each variable under study. All reported *p* values are two-sided with an alpha < 0.05 considered statistically significant. All analyses were performed using the IBM SPSS Statistics version 31.0.2.0.^1^

## 3 Results

### 3.1 Characteristics of the participants

Table 1 depicts characteristics of the entire subject sample as well as of the different analysis groups. The neurotypical children are presented and analyzed as a single group, rather than as three separate groups as in the original publication (20), after performing score comparisons of all questionnaires and subscales that demonstrated lack of differences between siblings of individuals with different NDDs (not shown). The grouping of all neurotypical participants allowed NDD-Neurotypicals comparisons with greater statistical power. Not displayed in Table 1 is the age range of the RTT participants older than 18 years, which corresponded to 18–20 years (mean 18.56, SD 0.46 years). Age was not significantly different among NDDs and Neurotypicals in the entire sample or in the age subsets. Sex distribution was comparable in the Neurotypical, AS, and PWS groups; however, consistent with the population, the RTT sample was almost exclusively female (99%). In the initial investigation, it was reported that most subjects (87%) were white. Table 2 depicts CSHQ, SBRD, and PDSS scores, including those from subscales, for the different age groups analyzed in the study.

**Table 1:** Characteristics of participants.

Sample characteristics	Neurotypicals	Angelman syndrome	Prader-Willi syndrome	Rett syndrome
N (% Female)	282 (54)	74 (57)	90 (57)	241 (99)
Age mean (SD) years	9.31 (4.34)	7.93 (3.71)	8.81 (3.94)	8.53 (3.86)
N 2–10 years	157	53	58	160
N 10–18 years	125	21	32	81
N ≥ 18 years*	N/A	N/A	N/A	15
N CSHQ	274	69	86	237
N SRBD	282	74	90	241
N PDSS (N valid age range**)	204 (191)	58 (51)	62 (60)	166 (163)

CSHQ, Children’s Sleep Habits Questionnaire; SRBD, Sleep-Related Breathing Disorder; PDSS, Pediatric Daytime Sleepiness Scale. *Excluded from total sample and sleep questionnaire counts. **Valid age range refers to the age range for which the scales have been validated. This varies across scales with the CSHQ being validated in ages 2–10, the SRBD being validated in ages 2–18, and the PDSS being validated in ages 5–17.

**Table 2:** Profiles of sleep questionnaire scores.

Age group	CSHQ Total	CSHQ bed-time resistance	CSHQ sleep onset delay	CSHQ sleep duration	CSHQ sleep anxiety	CSHQ night waking	CSHQ parasomnias	CSHQ disordered breathing	CSHQ daytime sleepiness	SRBD total	SRBD snoring	SRBD daytime sleepiness	SRBD inattention-hyperactivity	PDSS total
A Neurotypicals														
2–18 years	45.57 (10.53)	10.74 (1.78)	2.49 (0.74)	6.90 (0.65)	5.35 (1.78)	3.82 (1.29)	8.54 (1.77)	3.20 (0.69)	15.74 (2.68)	0.16 (0.15)	8.48 (1.38)	4.69 (1.09)	7.56 (1.94)	9.46 (5.75)
2–10 years	46.59 (11.53)	11.27 (1.71)	2.55 (0.69)	6.97 (0.48)	5.89 (1.91)	4.00 (1.48)	8.91 (1.92)	3.21 (0.51)	15.10 (2.61)	0.15 (0.14)	8.46 (1.26)	4.48 (0.89)	7.70 (1.83)	7.77 (4.89)
10–18 years	44.34 (9.07)	10.10 (1.66)	2.43 (0.79)	6.81 (0.80)	4.69 (1.33)	3.60 (0.98)	8.10 (1.45)	3.19 (0.85)	16.51 (2.57)	0.16 (0.16)	8.50 (1.51)	4.94 (1.26)	7.38 (2.05)	10.52 (6.02)
Cross-sectional (2–10 years vs. 10–18 years)	NS	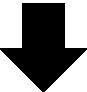 ***	NS	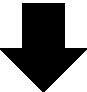 *	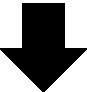 ***	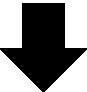 *	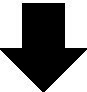 ***	NS	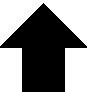 ***	NS	NS	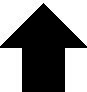 ***	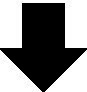 **	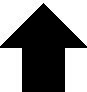 ***
Long. within-subject (12 month follow up)	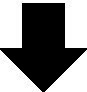 ***	NS	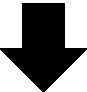 *	NS	NS	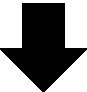 ***	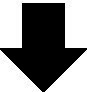 ***	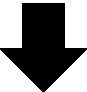 **	NS	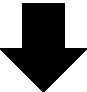 ***	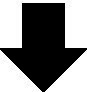 ***	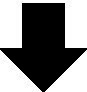 ***	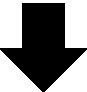 ***	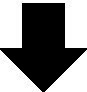 ***
B Angelman syndrome														
2–18 years	54.28 (11.12)	11.35 (1.82)	2.36 (0.69)	6.68 (0.72)	6.35 (1.85)	5.36 (1.65)	10.00 (1.69)	3.51 (0.89)	15.09 (3.01)	0.37 (0.13)	9.04 (1.68)	5.24 (1.30)	9.84 (1.66)	9.90 (4.30)
2–10 years	53.69 (10.79)	11.48 (1.83)	2.29 (0.71)	6.79 (0.68)	6.27 (1.80)	5.33 (1.71)	9.83 (1.40)	3.42 (0.68)	15.08 (3.30)	0.35 (0.12)	8.79 (1.55)	5.19 (1.29)	9.83 (1.81)	9.81 (4.37)
10–18 years	55.62 (12.02)	11.05 (1.80)	2.52 (0.60)	6.43 (0.75)	6.52 (1.99)	5.43 (1.54)	10.38 (2.20)	3.71 (1.23)	15.10 (2.28)	0.42 (0.13)	9.67 (1.88)	5.38 (1.36)	9.86 (1.28)	10.05 (4.28)
Cross-sectional (2–10 years vs. 10–18 years)	NS	NS	NS	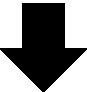 *	NS	NS	NS	NS	NS	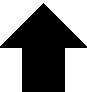 *	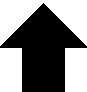 *	NS	NS	NS
Long. within-subject (12 month follow up)	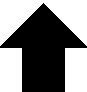 **	NS	NS	NS	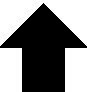 **	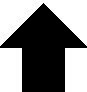 **	NS	NS	NS	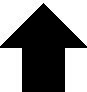 *	NS	NS	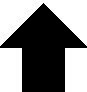 **	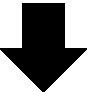 ***
C Prader-Willi syndrome														
2–18 years	44.02 (8.21)	10.26 (0.80)	2.98 (0.15)	6.88 (1.12)	4.45 (0.94)	4.74 (1.78)	9.05 (2.22)	3.40 (1.07)	12.71 (2.82)	0.32 (0.16)	9.41 (1.65)	5.26 (1.10)	8.97 (1.85)	10.18 (4.64)
2–10 years	43.63 (8.16)	10.24 (0.78)	2.98 (0.14)	6.91 (1.18)	4.43 (0.86)	4.85 (1.92)	9.15 (2.29)	3.41 (1.13)	12.48 (3.16)	0.31 (0.16)	9.31 (1.69)	5.14 (1.10)	8.98 (1.86)	8.93 (3.98)
10–18 years	44.69 (8.39)	10.28 (0.85)	2.97 (0.18)	6.84 (1.05)	4.50 (1.08)	4.56 (1.54)	8.88 (2.11)	3.38 (0.98)	13.09 (2.10)	0.34 (0.16)	9.59 (1.58)	5.47 (1.08)	8.94 (1.87)	11.34 (4.96)
Cross-sectional (2–10 years vs. 10–18 years)	NS	NS	NS	NS	NS	NS	NS	NS	NS	NS	NS	NS	NS	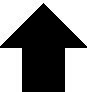 *
Long. within-subject (12 month follow up)	NS	NS	NS	NS	NS	NS	NS	NS	NS	NS	NS	NS	NS	NS
D Rett syndrome														
2–18 years	49.82 (10.58)	10.55 (1.23)	2.62 (0.67)	6.67 (1.03)	5.02 (1.42)	4.57 (1.43)	10.22 (1.91)	3.95 (1.38)	14.69 (2.98)	0.38 (0.16)	9.73 (1.91)	5.51 (1.38)	8.89 (1.99)	12.26 (5.16)
2–10 years	49.71 (11.07)	10.61 (1.30)	2.59 (0.70)	6.69 (1.11)	5.10 (1.46)	4.53 (1.46)	10.19 (1.87)	3.80 (1.24)	14.60 (2.94)	0.36 (0.16)	9.60 (1.91)	5.43 (1.34)	8.86 (1.99)	12.01 (4.95)
10–18 years	50.05 (9.64)	10.44 (1.07)	2.67 (0.61)	6.62 (0.86)	4.86 (1.31)	4.64 (1.37)	10.30 (2.00)	4.23 (1.58)	14.88 (3.05)	0.42 (0.15)	9.98 (1.90)	5.68 (1.44)	8.95 (2.02)	12.53 (5.39)
> 18 years	46.33 (9.27)	10.00 (0)	2.93 (0.26)	6.53 (1.25)	4.40 (0.63)	4.67 (1.50)	9.93 (1.28)	3.80 (0.94)	14.00 (2.36)	0.35 (0.14)	9.60 (1.45)	5.47 (0.92)	7.80 (1.97)	12.33 (3.15)
Cross-sectional (2–10 years vs. 10–18 years)	NS	NS	NS	NS	NS	NS	NS	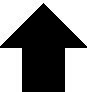 **	NS	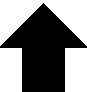 ***	NS	NS	NS	NS
Long. within-subject (12 month follow up)	NS	NS	NS	NS	NS	NS	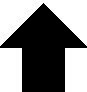 ***	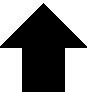 **	NS	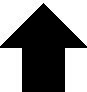 ***	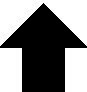 ***	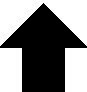 ***	NS	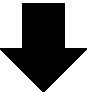 ***

All Means (SDs); CSHQ, Children’s Sleep Habits Questionnaire; SRBD, Sleep-Related Breathing Disorder; PDSS, Pediatric Daytime Sleepiness Scale; NS, No changes. *Bonferroni-adjusted Kruskal-Wallis or Wilcoxon Signed Rank *p* = 0.05–0.10. **Bonferroni-adjusted Kruskal-Wallis or Wilcoxon Signed Rank 0.01 > *p* < 0.05. *** Bonferroni-adjusted Kruskal-Wallis or Wilcoxon Signed Rank *p* < 0.01.

### 3.2 Profiles of sleep questionnaire scores in AS, PWS, and RTT

Analyses of baseline scores of scales and subscales across the NDDs, including only validation age-appropriate groups and employing Kruskal-Wallis tests followed by Bonferroni adjustments for multiple comparisons identified subscale profiles.

CSHQ: Children with AS or RTT showed significantly higher total CSHQ scores than Neurotypicals. The AS group had the highest scores while the PWS the lowest, which resulted in AS and RTT having higher scores than those with PWS and in statistical trend-level differences between AS and RTT (Table 3A). Analyses of the eight CSHQ subscales demonstrated that the differences in total scores between AS and RTT groups and the Neurotypicals reflected higher scores in Night Waking, Parasomnias, and Disordered Breathing. Several subscales showed lower scores in NDDs than in Neurotypicals including Bedtime resistance in PWS and RTT, Sleep onset delay in AS, Sleep anxiety in PWS and RTT, and Daytime sleepiness in PWS. Comparisons among NDDs reflected mainly the highest scores in AS and lowest scores in PWS. Exceptions to this pattern were found for the Sleep onset delay and Sleep duration subscales that displayed highest scores in PWS and, consequently, significant differences between PWS and AS or RTT. Sleep duration was the only CSHQ subscale without significant differences between Neurotypicals and NDD groups. Table 3A summarizes group comparisons of CSHQ scores.

**Table 3:** Differences in sleep questionnaire scores.

A CSHQ								
CSHQ total	CSHQ bedtime resistance	CSHQ sleep onset delay	CSHQ sleep duration	CSHQ sleep anxiety	CSHQ night waking	CSHQ parasomnias	CSHQ disordered breathing	CSHQ daytime sleepiness
AS*** > NT RTT*** > NT AS***, RTT*** > PWS AS* > RTT	PWS***, RTT*** < NT AS > PWS***, RTT**	PWS*** > NT AS** < NT PWS***, RTT*** > AS PWS*** > RTT	PWS*^ > RTT	PWS***, RTT*** < NT AS > PWS***, RTT** RTT** > PWS	AS***, PWS***, RTT*** > NT AS** > RTT	AS***, RTT*** > NT AS^ > PWS RTT*** > PWS	RTT*** > NT	PWS*** < NT AS***, RTT*** > PWS

B SRBD and PDS				
SRBD total	SRBD snoring	SRBD daytime sleepiness	SRBD inattention-hyperactivity	PDSS total
AS***, PWS***, RTT*** > NT RTT* > PWS	AS**, PWS***, RTT*** > NT RTT** > AS	AS***, PWS***, RTT*** > NT	AS***, PWS***, RTT*** > NT AS > PWS**, RTT***	RTT*** > NT RTT** > PWS

AS, Angelman syndrome; NT, Neurotypicals; PWS, Prader-Willi syndrome; RTT, Rett syndrome; CSHQ, Children’s Sleep Habits Questionnaire (2–10 years); SRBD, Sleep-Related Breathing Disorder (2–18 years); PDSS, Pediatric Daytime Sleepiness Scale (5–17 years). * Bonferroni-adjusted Kruskal-Wallis *p* = 0.05–0.10. **Bonferroni-adjusted Kruskal-Wallis 0.01 > *p* < 0.05. *** Bonferroni-adjusted Kruskal-Wallis *p* < 0.01. ^Significant on Benjamini-Hochberg-adjusted Mann–Whitney U test.

SRBD: All three AS, PWS, and RTT demonstrated significantly higher SRBD total scores than Neurotypicals. There were also trend-level higher total scores in RTT than PWS. NDD versus Neurotypicals comparisons of SRBD subscales showed a similar profile. However, comparisons among NDDs revealed different subscale patterns: significantly higher in RTT than AS for the Snoring subscale and significantly higher in AS than PWS and RTT for the Inattention-Hyperactivity subscale. No NDD differences were found for the Daytime sleepiness subscale (Table 3B).

### 3.3 Relationship between scales and subscales representing similar constructs

Disordered breathing: We compared the score profiles for the CSHQ Disordered breathing subscale, the SRBD total scale, and the SRBD Snoring subscale, the latter considered independently because of its core content. All three measures demonstrated similar higher RTT than Neurotypical scores, and SRBD Snoring scores were higher than in Neurotypical for the three NDDs. Higher scores in RTT than AS or PWS were also found for the SRBD measures. The three sets of scores demonstrated an overall agreement on highest severity in disordered breathing being present in RTT.

Daytime sleepiness: Contrasting disordered breathing measures, those evaluating daytime sleepiness showed an inconsistent pattern. While the PDSS exhibited highest scores in RTT (higher than Neurotypicals and PWS), the respective CSHQ and SRBD subscales showed highest scores in AS and, in the case of the CSHQ Daytime sleepiness, particularly high scores in Neurotypicals (Table 3). In order to better understand these profiles, we conducted Spearman correlations between items of PDSS and CSHQ Daytime sleepiness with comparable content (e.g., excluding sleepiness during specific activities covered only by the CSHQ). Spearman’s rho coefficients were > 0.6 for the entire sample and the within-group analyses for PDSS_item7 (How often does your child need someone to awaken him or her in the morning?) vs. CSHQ_item28 (Adults or siblings wake up child) and PDSS_item5 (How often does your child have trouble getting out of bed in the morning?) vs. CSHQ_item29 (Child has difficulty getting out of bed in the morning) but more variable, between 0.29–0.42, for PDSS_item4 (How often is your child tired and grumpy during the day?) vs. CSHQ_item31 (Child seems tired). Thus, the moderate-level correlation coefficients (60) in these analyses demonstrated relatively consistent responses among items that evaluate similar aspects of daytime sleepiness.

### 3.4 Changes in scores over time

Table 2 summarizes the outcome of analyses examining changes in scores over time. For this purpose, we used complementary cross-sectional and longitudinal approaches. Cross-sectional comparisons between 2–10 years (younger children) and 10–18 years (older children, adolescents) groups, for each condition and measure, revealed changes mainly in neurotypical participants. These consisted of cross-sectional score decreases in 5 CSHQ and 1 SRBD subscales and increases in the SRBD Daytime sleepiness and PDSS scales, which were partially matched by multiple decreases in the longitudinal analyses. The few changes observed for NDDs in the cross-sectional analyses contrasted with multiple significant results in the longitudinal analyses. Increases in scores for CSHQ Disordered breathing and SRBD total in RTT and for SRBD total in AS were the only findings observed in both types of analyses, highlighting the consistency of the disordered breathing data.

The availability of a small group of young adults (18–20 years) with RTT allowed a comparison with younger children and older children/adolescents groups within the NDD. The only measure that showed significant differences was the SRBD Inattention-Hyperactivity, higher in children/adolescents than adults. A few trend-level differences (*p* = 0.05–0.10) involved the CSHQ Bedtime resistance and Sleep anxiety subscales, with higher scores in younger children than adults, and the CSHQ Sleep Onset Delay that showed the opposite pattern. SRBD total scores were also higher in older children/adolescents than adults at the trend significant level.

## 4 Discussion

The present study intended to expand on an earlier evaluation of sleep difficulties in AS, PWS, and RTT, assessed by standardized questionnaires. We analyzed subscales of the CSHQ and SRBD and compared scores on these measures and the PDSS in a 12-month follow-up. Our goals were to characterize sleep problems of relatively greater severity in each NDD, to gain insight into their evolution, and to identify the most suitable measures for their evaluation. We found that night waking and snoring were problems present in all the NDDs, and parasomnias and daytime sleepiness affected both AS and RTT. Sleep questionnaire scores tended to decrease over time in neurotypical children, while they were more variable in their course in the NDDs, with increases over time of disordered breathing scores in AS and RTT. The high level of agreement among disordered breathing measures, and the relative stability in CSHQ scores in different age groups with the same condition, make them particularly suitable for multiple clinical and research applications.

Our earlier report of a high proportion (> 60%) of children with all three AS, PWS, and RTT having CSHQ total scores at the clinically significant problems level (20) did not specify which of the wide range of sleep difficulties covered by the measure contributed the most to these profiles. By comparing with neurotypical siblings, who represent the most adequate controls, the present study showed that significantly higher CSHQ Night waking scores were present in the three NDDs, and that they were more severe in AS. These findings supported previous publications reporting difficulties in sleep maintenance in the three NDDs (32–34, 43, 48, 50) and identify this sleep problem as a factor in AS overall more severe sleep disturbance (20). The lack of complementary measures of sleep maintenance, as those for disordered breathing and daytime sleepiness, makes follow up investigations necessary to further characterize this sleep problem. CSHQ and SBRD subscales evaluating snoring and disordered breathing also confirmed the literature, including the previous analysis of the RDCRN data (20), indicating that this is a sleep problem affecting all three AS, PWS, and RTT. Surprisingly, RTT and not PWS, long known to be associated with obstructive sleep apnea (27, 38, 40, 42), was the most severely affected NDD in terms of breathing abnormalities (22). Sleep-disordered breathing has been previously reported in RTT (49, 51). However, its magnitude and complexity have only been recently recognized. For instance, it has been found that obstructive sleep apnea is very common in RTT and that it is associated with changes in sleep architecture, higher anxiety, and more severe mood disturbances (52). Since awake breathing abnormalities attributed to central regulatory dysfunction are a major clinical manifestation in RTT (29, 30), the present data recommend additional research on the nature of sleep-disordered breathing in RTT. Our findings also support routine screening for obstructive sleep apnea in these NDDs.

Daytime sleepiness has been described in the three NDDs (19, 20, 34, 40, 50). Nevertheless, the addition of the CSHQ and SBRD Daytime sleepiness subscales to the previously reported PDSS only supported this being a major sleep problem in AS and RTT. Surprisingly, the CSHQ Daytime sleepiness subscale scores in PWS, a disorder linked to hypersomnia (41, 44), were lower than those of neurotypical subjects. This finding and the discordance between the PDSS and the CSHQ and SBRD subscales in terms of AS vs. RTT scores (i.e., higher for RTT in PDSS, higher for AS in CSHQ and SBRD) led to an examination of comparable items of the PDSS and the CSHQ, which revealed moderate correlations (60). Therefore, other aspects covered of these questionnaires, such as alertness latency or sleepiness during specific activities (e.g., watching TV) included only in the CSHQ (55), seem to be responsible for discrepancies within our results and with the literature. Nonetheless, our data support daytime sleepiness as a concern in both AS and RTT. Another pattern common to AS and RTT was higher scores than in Neurotypicals on the CSHQ Parasomnias subscale. This is not unexpected since night terrors have been described in AS (34, 42) and sleep laughing in RTT (48). These data may constitute the foundation for in-depth analyses of these complex sleep phenomena.

Other findings based on individual subscale scores that need additional investigation included in PWS higher scores on CSHQ’s Sleep onset delay, one of the two CSHQ subscales measuring difficulties initiating sleep, and on CSHQ’s Sleep duration than in Neurotypicals. Interestingly, several comparisons between the NDDs and the Neurotypical group demonstrated lower scores (i.e., lower severity) in the former. Specifically, PWS and RTT displayed lower scores on the CSHQ Bedtime resistance and Sleep anxiety subscales than Neurotypicals, and lower scores than Neurotypicals were also found for AS in the CSHQ Sleep onset delay subscale and for PWS on the CSHQ Daytime sleepiness subscale. The latter findings indicate that children with these NDDs do not demonstrate impairments in all aspects of sleep and that, for some difficulties, these may be lower than in typically developing children.

There are very limited longitudinal data on sleep difficulties in severe NDDs, including those in this study (48, 61–63). Evolution of sleep problems has been inferred from cross-sectional analyses (34, 44, 50). Longitudinal confirms different trajectories of sleep problems among these NDDs; while they are frequently present at early age in AS (62), only central sleep apnea is prevalent in infants with PWS (61, 63). RTT resembles AS in that multiple sleep problems are present since early ages (48). These few studies indicate that the sleep disturbances under evaluation tended to decrease in frequency or severity (48, 62, 63). Nonetheless, the fragmented nature of the data does not allow solid conclusions about any of the disorders. For these reasons, we intended to gain some insight into the evolution of sleep problems within the age range of the study by applying a dual strategy combining cross-sectional with longitudinal analyses. Neurotypical individuals demonstrated greater and more consistent changes; unaffected siblings displayed a decrease in scores on multiple sleep measures between early and late childhood/adolescence that was also detected over the 12-month period of longitudinal evaluation. Exceptions to this pattern were daytime sleepiness measures, which showed increases over time, and disordered breathing scales which did not display changes. Individuals with NDDs demonstrated multiple changes, mainly in the 12-month longitudinal analyses, without evident or consistent cross-sectional/longitudinal patterns other than increases in disordered breathing scores in RTT and AS. Inclusion in the cross-sectional analyses of a small group of young adults with RTT did not reveal any other changes in scores over time. At this point, adequately powered evaluations over longer periods of time, using the instruments we applied here as well as others, are necessary to determine the stability of sleep profiles in AS, PWS, and RTT. These data are essential for the planning of treatment trials in these disorders. The FREESIAS study in AS (64) is a good example of the type of investigation needed for these purposes. It consists of a prospective, longitudinal collection of patient- and caregiver-oriented clinical outcome assessments and biomarkers, including polysomnography, sleep actigraphy, and EEG, to identify clinical trial endpoints with adequate metric properties (64).

The initial (20) and current studies of the RDCRN data confirmed and expanded the sleep abnormality profiles of AS, PWS, and RTT (reviewed in (1, 6, 50). Sleep problems affected most children and adolescents with these NDDs, with sleep maintenance and disordered breathing abnormalities being common features. NDD-specific characteristics involved overall severity of sleep abnormalities as well as prominence of some of them. AS seems to be characterized by high severity of multiple sleep problems, including difficulties in sleep maintenance, sleep anxiety, parasomnias, daytime sleepiness, and disordered breathing, with the latter worsening over time. In line with their clinical phenotype similarities, RTT’s pattern resembles that of AS although its severity is milder and longitudinal worsening involves not only disordered breathing but, perhaps also, parasomnias (only cross-sectional data). PWS was the NDD with the lowest level of severity, including its distinctive sleep-disordered breathing, and the greatest score stability over time. The especially high scores on CSHQ Sleep onset delay suggest that difficulties with sleep initiation is a problem in PWS.

As mentioned above, our analysis of relationships between scales and subscales representing similar constructs showed high consistency among measures of sleep-disordered breathing but low agreement among those evaluating daytime sleepiness. This implies that selection of daytime sleepiness measures for clinical or research purposes should consider their differential content mentioned above and their use with complementary sources of information (e.g., caregiver interview). The CSHQ, the most widely used of the sleep questionnaires among other reasons because of its wide sleep problem coverage, appears to be a relatively stable measure within NDD from early childhood to early adulthood despite its validation limited to 2–10 years. Thus, in severe NDDs such as those under analysis here, the CSHQ could be applied to a wider age range than initially intended although data interpretation should be cautious. Incorporation of available instruments, as those used in this study, and future NDD-adapted measures to the growing field of intervention studies is imperative considering the association of sleep difficulties with behavioral and functional impairments in these populations (17, 19, 52).

Given the preponderance of sleep difficulties in NDD populations, together with limitations of caregiver reporting and self-report via questionnaires, there may be utility in using not only polysomnography (64, 65) but also newer generation wearable sensors to more objectively capture sleep quality and sleep-disordered breathing alongside formal sleep questionnaires and sleep diaries. In particular, wrist or finger-worn sensors that utilize photoplethysmography (PPG), which uses an infrared light to measure the volumetric variations of blood circulation, along with actigraphy tend to be more accurate with detecting wake time (66, 67). When obstructive sleep apnea is a concern, using a sensor that captures oxygen saturation (SpO2), and the addition of pulse oximetry (which captures SpO2) permits an assessment of hypoxemia and more closely approximates what is captured during gold-standard polysomnography. Also, given the concordance of sleep problems, autonomic dysfunction, and behavioral difficulties in these populations, it will be important to also consider the degree to which challenging behaviors exacerbate and/or contribute to sleep difficulties in future studies by additionally using behavioral questionnaires as well as possible physiological correlates that can be captured using wearable sensors (e.g., electrodermal activity, skin temperature, heart rate variability).

Limitations of this investigation were delineated in the initial publication on the dataset (20). In addition to those inherent to the more subjective sleep questionnaires (versus, for instance, polysomnography) and the lack of evaluation of factors potentially affecting sleep measure scores, such as comorbidities and medication use, the subject samples for the AS and PWS groups were relatively small. Moreover, only sleep-disordered breathing and excessive daytime sleepiness were assessed with multiple measures. Although planned as an exploratory analysis, the use of the CSHQ in participants aged 10–18 years, beyond its originally validated age range, should be considered a limitation of this investigation. The longitudinal data was actually serial, involving two time points separated by only 12 months and small samples without individuals with PWS. Despite all these shortcomings, the present study provides the basis for a more informed management of sleep difficulties in AS, PWS, and RTT contributing to the delineation of basic profiles of each condition. Furthermore, it provides the foundation for follow-up studies, including those employing objective methods, assessing evolution and response to current and new treatments of sleep disorders in severe NDDs.

## 5 Conclusion

The present study expanded our current understanding of sleep abnormalities in AS, PWS, and RTT by demonstrating common problems, such as difficulties with sleep maintenance and disordered breathing, and identifying NDD-distinctive problems. Severe sleep-disordered breathing is characteristic of RTT while parasomnias and daytime sleepiness are distinctive of both AS and RTT. Sleep problems have a variable course in these NDDs, although disordered breathing seems to increase over time in AS and RTT. The high level of agreement among disordered breathing measures, and the relative stability of CSHQ scores, among these severe NDDs, make them suitable measures for clinical and research purposes. These findings may also be consistent with the notion that sleep problems tend to be more prominent in disorders with greater overall clinical impairment, although clinical severity was not directly assessed in the present study. In addition, the data show that evolution of sleep disturbances in complex NDDs is less predictable or more difficult to evaluate than that of neurotypical children. For all these reasons, characterization of sleep problems in NDDs should be prioritized and involved more targeted measures including more objective and refined instruments. These steps are necessary to ensure improvements in the clinical management of sleep difficulties and their proper inclusion in clinical trials.

## Data Availability

Publicly available datasets were analyzed in this study. This data can be found at: the datasets from the Angelman, Rett and Prader-Willi Consortium have been deposited to the database of Genotypes and Phenotypes (dbGAP) repository per a predefined schedule at regular intervals (https://www.ncbi.nlm.nih.gov/projects/gap/cgi-bin/study.cgi?study_id=phs000574.v1.p1).

## Footnotes

1
https://www.ibm.com/products/spss-statistics
