# Supplementary material for: Profiles of sleep disturbances in Angelman, Prader-Willi, and Rett syndromes: analysis of standardized questionnaires
Source: Front Neurol. 2026 Jul 22;17:1854819. doi: 10.3389/fneur.2026.1854819 (PMC13437362; doi:10.3389/fneur.2026.1854819)
Supplement: Supplementary file 1 [file Data_Sheet_1.PDF]

SUPPLEMENTARY MATERIAL

Eligibility Criteria (https://clinicaltrials.gov)

NCT00296764 – Angelman syndrome

Inclusion Criteria:

- Molecular diagnosis of Angelman syndrome OR
- Meets all major diagnostic criteria for Angelman Syndrome and 3 of the 6 minor criteria:

Major Criteria:

- Functionally severe developmental delay
- Speech impairment; none or minimal words used
- Movement or balance disorder
- Behavioral uniqueness, frequent laughs/smiling, excitable personality, hand flapping, short attention span

Minor Criteria:

- Deceleration in head circumference growth (post-natal)
- Seizures (myoclonic, absence, drop, tonic-clonic)
- Abnormal EEG (with patterns suggestive of AS, or hypsarrhythmia)
- Sleep disturbance
- Attraction to or fascination with water
- Drooling

Exclusion Criteria:

- Does not meet diagnostic criteria for Angelman Syndrome
- Other medical or genetic disorders (except autism)
- Born extremely premature

Study Population

- Patients with Angelman syndrome (molecular or clinical diagnosis) between the ages of 1 day and 60 years.

NCT03718416 – Prader-Willi syndrome

Inclusion Criteria:

- Confirmed diagnosis of PWS
- At least 5 years of age
- Live in the United States, Canada, Australia, or New Zealand

- Must be enrolled or willing to enroll in the Global PWS Registry (https://pwsregistry.org)

Participants or their caregiver must also meet all of the following criteria:

- Have access to the internet to complete online surveys at least every 6 months
- Agree to receive reminders to complete online surveys
- Allow the staff to ask questions about survey responses if more information is needed and to enter data into the surveys

Exclusion Criteria:

- Is not able to read and understand English

Study Population

- Participants with PWS who are at least 5 years old and live in the United States, Canada, Australia, or New Zealand.

NCT02738281 – Rett syndrome

Inclusion Criteria:

- Individuals of both genders and of all ages, with RTT, MECP2 Dup, and, RTT-related disorders including those with mutations or deletions in CDKL5 and FOXG1 genes, or those with RTT (atypical or typical) who are mutation negative.

Exclusion Criteria:

- Individuals who do not meet the above criteria will be excluded.

Study Population

- Females and males of all ages must have complete testing for MECP2, FOXG1 and CDKL5 genes mutations AND must meet these requirements:
  Gene positive for a sequence mutation, duplication or deletion in one of these 3 genes.
  OR Meet consensus criteria for Rett syndrome (typical or atypical).
